# Correction: Causal role of the dorsolateral prefrontal cortex in modulating the balance between Pavlovian and instrumental systems in the punishment domain

**DOI:** 10.1371/journal.pone.0295458

**Published:** 2023-11-30

**Authors:** Hyeonjin Kim, Jihyun K. Hur, Mina Kwon, Soyeon Kim, Yoonseo Zoh, Woo-Young Ahn

The first author, Hyeonjin Kim, is incorrectly noted as the corresponding author. The correct corresponding author is Woo-Young Ahn. Woo-Young Ahn’s email address is: wahn55@snu.ac.kr.
