# Supplementary figures and images for: Causal role of the dorsolateral prefrontal cortex in modulating the balance between Pavlovian and instrumental systems in the punishment domain
Source: PLoS One. 2023 Jun 2;18(6):e0286632. doi: 10.1371/journal.pone.0286632 (PMC10237433; doi:10.1371/journal.pone.0286632)

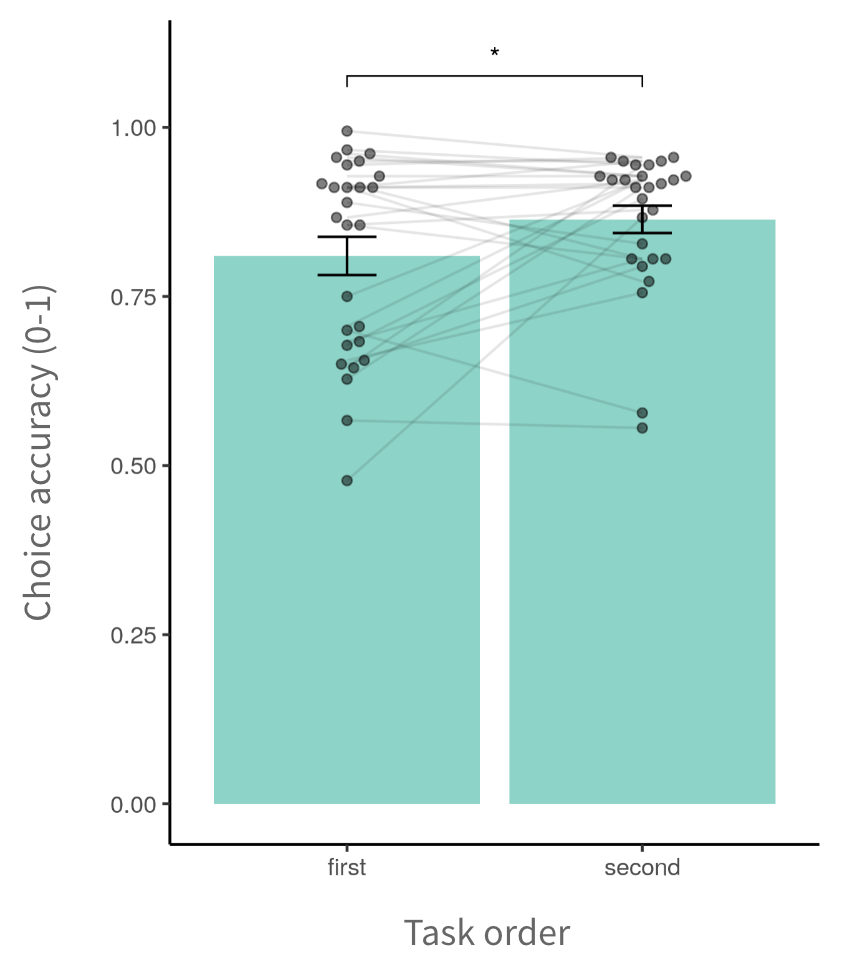

Supplement: S1 Fig — When comparing the task performance of the first (third day) and the second time (sixth day) the participants conducted, we found significantly higher accuracy in the second time. This indicates that the second task performance is vulnerable to confounding effects caused by the task order. Therefore, we included only the first task performance to eliminate this potential confounding factor. Error bars indicate SEM. *p < 0.05. (TIFF) [file pone.0286632.s001.tiff]

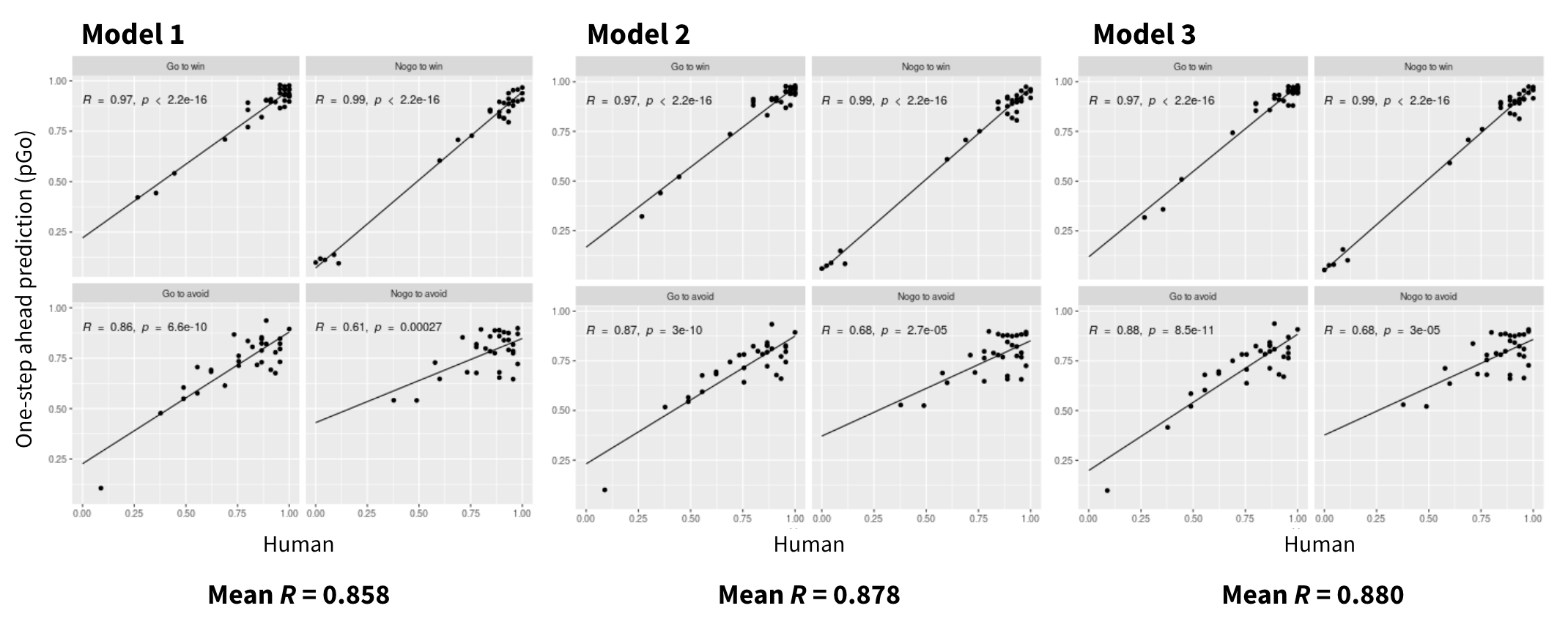

Supplement: S2 Fig — We conducted the one-step-ahead prediction to compare the predicted results and actual choice behaviors. To generate predictions, we utilized 8000 MCMC samples (2000 samples x 4 chains) drawn from individual posterior distributions to predict the probability of a “Go” choice in each of the four conditions, and averaged the predictions with each participant. Model 3 (7-parameter model) emerged as the best performing model, with a mean correlation coefficient of 0.890. (TIFF) [file pone.0286632.s002.tiff]

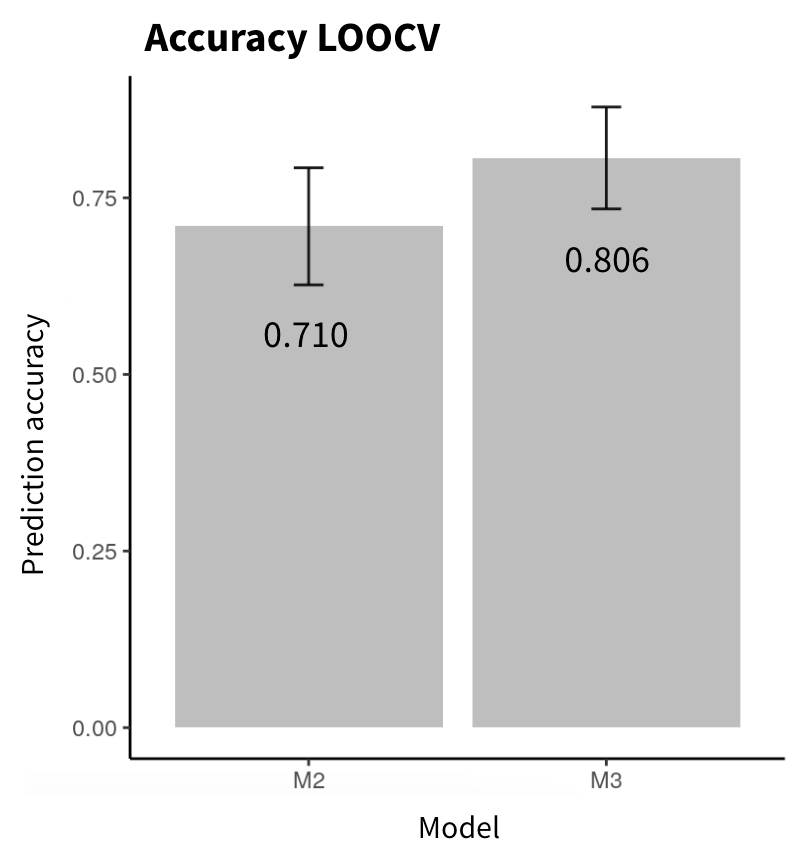

Supplement: S3 Fig — Although Model 3 was the best model, Model 2 also showed closely comparable prediction values (mean correlation value = 0.885). The only difference between the two models is the utilization of separate Pavlovian bias parameters for reward and punishment. Consequently, we further compared Models 2 and 3 and investigated whether having two parameters for Pavlovian bias (as in Model 3) would aid in classifying tDCS group membership. By employing ridge logistic regression and leave-one-out cross-validation, we compared the classification accuracy of both models. Model 3 (AUC = 0.806) demonstrated enhanced classification accuracy in comparison to Model 2 (AUC = 0.710). Error bars indicate SEM. (TIFF) [file pone.0286632.s003.tiff]
