# Supplementary material for: Causal role of the dorsolateral prefrontal cortex in modulating the balance between Pavlovian and instrumental systems in the punishment domain
Source: PLoS One. 2023 Jun 2;18(6):e0286632. doi: 10.1371/journal.pone.0286632 (PMC10237433; doi:10.1371/journal.pone.0286632)
Supplement: S1 Table — (DOCX) [file pone.0286632.s004.docx]

**S1 Table**. Side effects

|  | Sham  (# session = 42) | Anode  (# session = 51) | Total  (# session = 93) | p-value |
| --- | --- | --- | --- | --- |
| tingling | 0.714 (0.944) | 1.176 (1.126) | 0.968 (1.068) | 0.037 |
| itching | 0.738 (1.037) | 0.824 (1.178) | 0.785 (1.112) | 0.714 |
| skin irritation | 0.690 (1.137) | 0.490 (0.946) | 0.581 (1.035) | 0.356 |
| skin pain | 0.405 (0.665) | 0.373 (0.747) | 0.387 (0.708) | 0.828 |
| headache | 0.310 (0.780) | 0.000 (0.000) | 0.140 (0.544) | 0.006 |
| fatigue | 1.333 (1.391) | 1.098 (1.063) | 1.204 (1.221) | 0.358 |
| difficulty  concentration | 1.405 (1.466) | 0.843 (1.046) | 1.097 (1.277) | 0.034 |
| mood  disturbance | 0.119 (0.328) | 0.196 (0.601) | 0.161 (0.495) | 0.459 |
| visual  distortion | 0.333 (0.786) | 0.157 (0.505) | 0.237 (0.649) | 0.194 |

* scale 0 - 5
