# Supplementary material for: Causal role of the dorsolateral prefrontal cortex in modulating the balance between Pavlovian and instrumental systems in the punishment domain
Source: PLoS One. 2023 Jun 2;18(6):e0286632. doi: 10.1371/journal.pone.0286632 (PMC10237433; doi:10.1371/journal.pone.0286632)
Supplement: S2 Table — (DOCX) [file pone.0286632.s005.docx]

**S2 Table**. Manipulation check of double-blind design

|  | **Sham**  **(# session = 42)** | **Anode**  **(# session = 51)** | **Total**  **(# session = 93)** | **p-value** |
| --- | --- | --- | --- | --- |
| continuity |  |  |  | < 0.001 |
| continue | 4 (9.5%) | 22 (43.1%) | 26 (28.0%) |  |
| discontinue | 38 (90.5%) | 29 (56.9%) | 67 (72.0%) |  |
| duration |  |  |  | < 0.001 |
| less than 5 mins | 30 (79.0%) | 16 (55.2%) | 46 (68.7%) |  |
| less than 10 mins | 6 (15.8%) | 8 (27.6%) | 14 (20.9%) |  |
| less than 15 mins | 2 (5.3%) | 5 (17.2%) | 7 (10.5%) |  |
| direction |  |  |  | 0.47 |
| toward cheek | 16 (38.1%) | 25 (49.0%) | 41 (44.1%) |  |
| far from cheek | 8 (19.0%) | 6 (11.8%) | 14 (15.1%) |  |
| don't know | 18 (42.9%) | 20 (39.2%) | 38 (40.9%) |  |

* Continuity: did you feel that the electric current continued to flow during the tDCS experiment?

* Duration: if not, how long did you feel that the electric current flowed?

* Direction: did you feel that the current flowed toward your cheek or far from your cheek?
