# Supplementary material for: Causal role of the dorsolateral prefrontal cortex in modulating the balance between Pavlovian and instrumental systems in the punishment domain
Source: PLoS One. 2023 Jun 2;18(6):e0286632. doi: 10.1371/journal.pone.0286632 (PMC10237433; doi:10.1371/journal.pone.0286632)
Supplement: S1 File — Includes information on electric shock protocol. (DOCX) [file pone.0286632.s006.docx]

**Electric shock**

The electric shock was tailored to each participant at a level of ‘moderately unpleasant’ for safety. Each participant conducted a task consisting of 3 blocks to find the level. In the first block, electric stimulation of direct currents below 7 mA was first applied, and participants rated their subjective feelings on an 11-point scale (0 = not unpleasant at all, 10 = very unpleasant). If the participants rated it as 6-10 points, the intensity of the next stimulation decreased, otherwise increased. The amount of change in stimulation was halved each time it changed. When the amount of change was less than 0.1 mA, the first block ended [[1–5]](https://paperpile.com/c/CBwJ4k/dgYP+dYZM+niTD+HeUn+Gvks). The second and third blocks had the same process as the first, and only the currents initially given were less than 6 mA and less than 8 mA, respectively. By substituting a series of electrical stimulation intensities into the sigmoid function, the intensity corresponding to the subjective intensity of 5 points (moderately unpleasant) was estimated. No matter what, the current higher than 12.4 mA was not used throughout the process [[6]](https://paperpile.com/c/CBwJ4k/Zm7T). The participants were fully informed that they can stop the process at any time (i.e. discomfort during the electrical stimulation experiment).

**Reference**

1. [Cornwell BR, Echiverri AM, Covington MF, Grillon C. Modality-specific attention under imminent but not remote threat of shock: evidence from differential prepulse inhibition of startle. Psychol Sci. 2008;19: 615–622.](http://paperpile.com/b/CBwJ4k/dgYP)

2. [Crockett MJ, Kurth-Nelson Z, Siegel JZ, Dayan P, Dolan RJ. Harm to others outweighs harm to self in moral decision making. Proc Natl Acad Sci U S A. 2014;111: 17320–17325.](http://paperpile.com/b/CBwJ4k/dYZM)

3. [Mobbs D, Petrovic P, Marchant JL, Hassabis D, Weiskopf N, Seymour B, et al. When fear is near: threat imminence elicits prefrontal-periaqueductal gray shifts in humans. Science. 2007;317: 1079–1083.](http://paperpile.com/b/CBwJ4k/niTD)

4. [Phelps EA, Delgado MR, Nearing KI, LeDoux JE. Extinction learning in humans: role of the amygdala and vmPFC. Neuron. 2004;43: 897–905.](http://paperpile.com/b/CBwJ4k/HeUn)

5. [Vlaev I, Seymour B, Dolan RJ, Chater N. The price of pain and the value of suffering. Psychol Sci. 2009;20: 309–317.](http://paperpile.com/b/CBwJ4k/Gvks)

6. [Rhudy JL, Meagher MW. Fear and anxiety: divergent effects on human pain thresholds. Pain. 2000;84: 65–75.](http://paperpile.com/b/CBwJ4k/Zm7T)
